# Supplementary material for: Asymmetric changes in foot anthropometry with pregnancy may be related to onset of lower limb and low back pain
Source: PLoS One. 2024 Feb 23;19(2):e0292115. doi: 10.1371/journal.pone.0292115 (PMC10890715; doi:10.1371/journal.pone.0292115)
Supplement: Attachment — Submitted filename: Responses to Reviewers FINAL submitted.pdf [file pone.0292115.s002.pdf]

Responses to Reviewers:

We are thankful to the reviewers for their detailed read-through of our manuscript and their insightful input. We appreciate their comments and have done our best to address them. Our responses to their comments are shown below in indented italics following each of their requested revisions.

Reviewer 1:

Reviewer #1: This manuscript is titled "Asymmetric changes in foot anthropometry with pregnancy may be related to onset of the lower limb and low back pain". There is a great insight into investigating foot asymmetry and pain in pregnancy.

However, you mentioned in the abstract section that the purpose of this article was to compare asymmetric alignment in females with and without pain during pregnancy and to assess the relationship between asymmetric alignment and pain severity, and in the results section, you have mentioned that the "first aim" and the "second aim", we did not understand what you mean by your purpose. We can't quite understand the purpose of this manuscript. Does the author want to investigate the symmetric alignment in females with and without pain during pregnancy and to assess the relationship between asymmetric alignment and pain severity or does the author want to quantify foot asymmetries in females with pain during pregnancy compared to pregnant females without pain and a control group of never pregnant females? These two purposes are different, but important for this research, and we suggested the author revise them clearly in the article.

> *Thank you for noticing that the purpose stated in the abstract of our original submission did not align with what was done in the study. Therefore, we amended the purpose in the abstract to read as follows: "The* ***purpose*** *of this study was twofold: 1) to compare asymmetric alignment in pregnant females with and without pain during pregnancy and in nulliparous controls and 2) to assess the relationship between asymmetric alignment and pain severity in all participants."*

We consider the results section of this manuscript mentioned that pain during pregnancy can be predictive of pain post-partum, how to conclude this viewpoint? In this research or previous report? Please provide relevant reference documentation.

> *We have added reference that state the pain in pregnancy is predictive of pain post-partum in the final paragraph of the discussion on line 322.*

We did not understand the result that pain during pregnancy can be predictive of pain post-partum, the participants in this manuscript concluded ten pregnant females in their third trimester and nine nulliparous controls, how to get the relationship between these two groups?

> *We agree that our study cannot report this because we did not assess pain post-partum. Rather, our purpose was to reiterate the relevance of our study in relating pain to malalignment. If pain during pregnancy was short term and resolved shortly after delivery, this pain would be of little concern. However, the pain is a long-term issue for many women. Our purpose was to examine alignment and pain and this is important*

because this pain could be predictive of pain post-partum, and we now cite the statements about this relationship.

The sample size used in this article is small. Is its between-group analysis statistically significant? Please explain this clearly to the author. Why such an analysis and comparison if it doesn't make much sense?

On line 214, we state that none of the between-group analyses found statistical significance for any variable tested.

At the same time, the sample size of the subjects mentioned by the author in the limitations, which is also the concern of the reviewer

We acknowledge that our sample size is small. We actively searched for participants for several months. We live in a small rural college town and our sample size was limited by participant availability and willingness to participate. In the end, we needed to complete this study in order for the first author on the study to complete her degree requirements and graduate from our MS program. We did not continue the study because we did not want a second investigator taking the alignment measurements due to inter-tester variability.

Reviewer #2:

This manuscript entitled "Asymmetric changes in foot anthropometry with pregnancy may be related to onset of lower limb and low back pain" primarily aimed to investigate the relationship between asymmetric in lower extremity alignment or foot posture and pain severity. The results of this study provide guidance for public health and bioengineering. While it is a very interesting topic. But I think this manuscript has some flaws to fill in before it can be published in a journal. There are several questions should be addressed, which list below. I give a major revision for this manuscript.

Specific comments

In the abstract part. In the opinion of reviewer, the author provided too much background descriptions in this part, which may be too long-winded. I suggest that the authors provide more detailed descriptions of the results and conclusions of this study in this part.

We shortened the detailed of the methods and added the following sentence to the results section of the abstract: "No statistical differences (p>0.05) were found between pregnant females with or without pain and controls for any of the metrics."

"Ten pregnant females in their third trimester and nine nulliparous controls participated. Informed consent was obtained." The authors recruited only 10 pregnant females in their trimester and none nulliparous contrils participated. How was this sample size calculated?(Line 43-44)

This was a sample size of convenience. We had hoped to be able to recruit more participants but were unable to find pregnant women in their third trimester who were willing to come in to for testing. We had to close enrollment for this study in order for

the first author to complete her degree requirements and graduate from our MS program. We did not continue the study because we did not want a second investigator taking the alignment measurements because no one else in the lab group was trained to measure these measurements yet.

In the Introduction part. “Approximately 50% of pregnant females report low back and lower extremity pain during pregnancy, with nearly a quarter reporting severe pain reducing overall quality of life” While the Ostgaard 1991 and Vullo 1996 references are seminal, there are more recent publications that discussed the low back and lower extremity pain of pregnant females. Please provide the latest references in this sentence. (Line 63-64)

We have added more recent references: Kesikburun et al 2018; Gutke et al 2017; Weis et al 2018; and more recent reference for post-partum Tavares et al 2020

“thus the purpose of this study was to assess asymmetries between left and right limbs in foot length (FL), foot width (FW), arch index (AI), arch rigidity index (ARI), arch height index (AHI), arch drop (AD), rearfoot angle (RFA), and pelvic obliquity (PO), as well as overall joint laxity during pregnancy.” On what basis did the authors choose these variables. (Line 81-83)

We selected these variables because 1) they have been reported previously in the literature as being related to body alignment and/or pain and 2) we had the capability to assess them in our laboratory. These have been reported by prior authors cited in our introduction, primarily Damen (20) and Harrison (18).

In the reviewer’s opinion, the authors’ description of bilateral asymmetry is not complete enough, I suggest that the authors add more descriptions about the biomechanical asymmetry in pregnant female.

To address this concern, we specifically added that our “asymmetry” is defined as the absolute difference between the right and left sides on lines 87 and 135.

In the Methods part. “Nineteen healthy females ages 21-34 participated in this study.” How was this sample size calculated? (Line 99-100)

Please see our earlier comment on this. This was a sample size of convenience. However, even though the sample size was small, we did find statistical significance in our 2nd aim: to quantify relationships between asymmetry and pain.

The reviewer recommended that the authors provide one or more citations to support the recruitment and screening criteria of participants in this study. (Line 99-100)

We have added references to our exclusion criteria to support their validity in our study. We selected these criteria due to their effect on pain e.g. scoliosis and injury may increase self-reported pain, but diabetes and other forms of neuropathy may decease self-reported pain.

In the reviewer's opinion, the authors provide a lot of detailed procedures and stutistics, which makes the description too lengthy. To make the process clearer to the reader, it is recommended to add a flowchart. (Line 100-105)

Thank you for this suggestion. We have included a flow chart as figure 1.

9. In the results part, in the opinion of the reviewer, the pixel in Figure 1 is too blurred, please replace a clearer figure.

We have replaced this with a higher quality image.

10. In the discussion part, “Because arch rigidity is based on the flexibility of the foot, it is important to note that these measures could also be affected by swelling.” Please add a reference to support this sentence. (Line 255-257)

We have rewritten this paragraph and this sentence no longer exists in its same form. However, the concept is still in that paragraph. We cite Alvarez et al (16) for the concept that swelling may affect arch due to a ‘bottoming out’ of the arch due to edema.

11. What are the limitations of this study? Please provide relevant description.

In our prior submission, the study limitations were interspersed throughout the discussion. However, we agree with your suggestion to create a separate paragraph to address specific limitations. Therefore, we have reworked the discussion and now include a limitations section.

12. In the Conclusion part. In the opinion of the reviewer, the description in the conclusion part was too verbose, and the reviewer suggests that the authors should abbreviate the section and focus on the main findings of this study.

We have reworked this section to omit material that may not be as relevant here and focus on the main findings.
