# Supplementary material for: Asymmetric changes in foot anthropometry with pregnancy may be related to onset of lower limb and low back pain
Source: PLoS One. 2024 Feb 23;19(2):e0292115. doi: 10.1371/journal.pone.0292115 (PMC10890715; doi:10.1371/journal.pone.0292115)
Supplement: Attachment — Submitted filename: Responses to reviewers comments 2.1 submitted.docx [file pone.0292115.s003.docx]

We would like to thank the Reviewers and the Editor for their input into this manuscript. The Editor asked us to provide enough data to answer the limited number of subjects. Reviewer 1 listed several issues that render the study, in their opinion, unpublishable. We have provided rebuttals to these issues below. Editor and Reviewer comments are shown in regular black font while our rebuttals are shown indented in blue font.

Editor Comment:

The authors shall provide enough data to answer the limited number of subjects.

We agree that our small sample size is a major limitation of this study. However, we feel that our study has merit and provides valuable information to clinicians and others who study pain, or the prevention of pain, in pregnancy. This was mentioned on line 311 where we mention sample size being small is a limitation.

We addressed that the significant results may be limited by sample size, referring to the correlations in our Specific Aim 2. With regard to Specific Aim 1 in which we compared groups using an ANOVA, nothing was significant. We mention this in the results and then we discuss effect sizes in the discussion which are independent of sample size. We make no conclusions about those results - only the correlations (Specific Aim 2). On lines 250-251, we added the statement: “In this study, we found no significant differences in FW asymmetries between groups; however, sample size was small and calculating effect sizes provided some insight into trends. There was a large effect size (η2=0.17) in which pregnant females who reported pain trended towards having greater FW asymmetries than those without pain and controls.”

Reviewer 1’s Comments:

First off, the artificial measurement of foot characteristics has a significant inaccuracy. Using a foot scanner could be more objective in this case, in my opinion.

We acknowledge that medical imaging may provide more accurate data than the Arch Height Index and Arch Index. However, we believe that these measures are useful tools in their own right with sufficient validity for the current study.

The Arch Height Index and Arch Index are widely used measurements in biomechanics research. A search of GoogleScholar with the keywords “Arch Height Index” reveals 988 publications which include this measurement. In specifically looking at pregnancy, a GoogleScholar search of “Arch Height Index” and “pregnancy” found 71 publications on the topic. Similarly, a search of GoogleScholar with the keywords “Arch Index” results in 4,040 publications using this measure. If “Pregnancy” is added as a search term, then 212 publications are listed as having Arch Index and pregnancy in them. Given that so many peer-reviewed publications have used these tools, we assert that the biomechanics scientific community accepts these methods as valid.

Arch Height Index: Butler et al. (2008) established the reliability (ICC) of the AHI to be 0.94. GoogleScholar metrics report that this reference article has been cited by 179 other publications. The Weimar and Shroyer (2013) publication which reports normative AHI values for young women has been cited in 29 publications.

Arch Index: The seminal publication defining Arch Index was by Cavanagh and Rodgers (1987). This work has been cited in 949 subsequent publications that have used this measure. McCrory et al. (1997) reported a correlation of r=0.67 between footprint obtained Arch Index and true navicular height obtained via a radiograph. Chu et al. (1995) reported a correlation of r=0.70 between arch index obtained via a digital image and that obtained via footprint.

We acknowledge that lower extremity edema associated with pregnancy will have a negative effect on the accuracy of Arch Height Index and Arch Index. However, we included this as a limitation of the study in our manuscript.

References:

Butler RJ, Hillstrom H, Song J, Richards CJ, and Davis IS. Arch Height Index Measurement System: Establishment of Reliability and Normative Values. Journal of the American Podiatric Medical Association. 98(2): 102-106, 2008.

Cavanagh PR and Rodgers MM. The arch index: A useful measure from footprints. Journal of Biomechanics. 20(5): 547-551, 1987.

Chu WC, Lee SH, Chu W, Wang TJ, Lee MC. The use of arch index to characterize arch height: a digital image processing approach. IEEE Transactions on Biomedical Engineering. 42(11): 1088-1093, 1995.

McCrory JL, Young MJ, Boulton AJM, and Cavanagh PR. Arch index as a predictor of arch height. The Foot. 7(2): 79-81, 1997.

Weimar WH and Shroyer JF. Arch Height Index Normative Values of College-Aged Women Using the Arch Height Index Measurement System. Journal of the American Podiatric Medical Association. 103(3): 213-217, 2013.

Additionally, the VAS pain is only suitable for supplemental reference. If conclusions are drawn using this data as the benchmark, it is insufficient.

Hunt and Hurwit (2013) did a systematic review on all clinical articles reporting on foot and ankle pain in six orthopedic journals over a ten-year period. They concluded that the American Orthopaedic Foot and Ankle Society Scale (AOFAS) was the most common measure used for pain, as it was included 55.9% of the 878 publications they reviewed. The VAS scale was the second most commonly used pain scale, as it was used in 22.9% of the studies. The VAS Foot and Ankle differs from the AOFAS score in that the VAS is a survey of perceived pain and function, whereas the AOFAS score requires the investigator to perform a physical examination of the subject. Other scales of pain and function include the Ankle Osteoarthritis Score (AOS) and the Foot Function Index (FFI). The AOS scale of lower extremity pain is only validated in patients with ankle OA and would not be valid for use in our study (Domsic and Saltzman, 1998). Similarly, the FFI is validated for low functional individuals with foot disorders or patients with arthritis or foot and ankle problems and is not considered appropriate for higher-functioning individuals (Agel et al, 2005). The VAS Foot and Ankle scale that we used in this study was validated for use in a variety of pathological conditions (Stuber et al., 2011).

The Visual Analog Scale (VAS) is frequently employed in biomechanics research. A search of GoogleScholar for “visual analog scale” and “biomechanics” yielded more than 24,300 biomechanics-related publications that use the VAS as a measure of subject perception of pain. A GoogleScholar search using the keywords “VAS Foot and Ankle” and “biomechanics” resulted in 37 biomechanics studies that used the VAS Foot and Ankle to evaluate subject pain.

Given the number of studies who have also used the VAS Foot and Ankle and the fact that it is a survey-based scale of perceived pain and is not based on a physical assessment, we determined that the VAS Foot and Ankle was the best scale to use for our study.

Kelly (2001) determined that clinically-significant patient-reported pain improvement or worsening corresponded to a change of ~1.2 on the VAS scale across three groups of subjects who reported “mild”, “moderate”, and “severe” pain, respectively. We used a VAS score of 3 as the delineation between our “pregnant pain” and “pregnant no pain” groups, in accordance with Kelly (2001).

We acknowledge that pain-level is subjective and is based on each subject’s perception of their discomfort. The validity of the VAS for use in pain measurement has been questioned. Landy and Sheppeard (1985) asserted that the results of the VAS are non-linear and prone to bias and thus it should not be used as a serial measure of pain severity. We did not use the VAS as a serial measure. Our participants each made one visit for the study.

References:

Agel J, Beskin JL, Brage M, et al. Reliability of the foot function index: a report of the AOFAS outcomes committee. Foot and Ankle International. 26: 962-967, 2005.

Domsic RT and Saltzman CL: Ankle osteoarthritis scale. Foot and Ankle International. 19: 466-471, 1998.

Hunt KJ and Hurwit DBA. Use of patient-reported outcome measures in foot and ankle research. The Journal of Bone and Joint Surgery. 95(16): e118, 2013.

Kelly AM. The minimum clinically significant different in visual analogue scale pain score does not differ with severity of pain. Emergency Medicine Journal. 2001. http://dx.doi.org/10.1136/emj.18.3.205

Langley GB and Sheppeard H. The visual analogue scale: its use in pain measurement. Rheumatology International. 5: 145-148, 1985.

Stuber J, Zech S, Bay R, Qazzaz, A, and Richter M. Normative data of the Visual Analogue Scale Foot and Ankle (VAS FA) for pathological conditions. Foot and Ankle Surgery. 17(3), 166-172, 2011).

Second, the sample size used in this research is too little to adequately support the results. Only 5 women who were pregnant in all experienced this kind of agony.

Our results were not significant thus we did not discuss them as such. We mentioned trends based on effect sizes and made no conclusions regarding this aim. We simply stated we needed a larger sample size to be able to detect differences in alignment that may exist – line 330 we said “differences in alignment that *may* exist”.

And this topic should be treated differently for pregnant women. Pregnancy timing is also significant. This was not considered by the writers.

We do not understand this comment. What topic specifically: comparing asymmetry in pregnant women who report pain, those who do not report pain, and non-pregnant controls, or looking at the relationship between asymmetry magnitude and pain level? We do not understand what is meant by “treated differently for pregnant women”.

We also do not understand what is meant by “pregnancy timing”. All of our pregnant participants were in their third trimester. The subjects in the pregnant pain group were at 31.0 ± 1.6 weeks pregnant while the subjects in the pregnant no-pain group were at 32.0 ± 2.0 weeks pregnant. Thus, our pregnant participants were all within a few weeks of each other. These data are provided in Table 1.

Last but not least, if the author intends to assess the relationship between bone discomfort in pregnant women and foot form characteristics. I believe this research needs further validation.

We apologize, but we do not understand this question. We do not use the word “bone” or name one of the lower extremity bones (femur, tibia, fibula, calcaneus, etc.) as a site of pain or discomfort in the submitted manuscript. We assessed regional pain in the lower back, hip/buttocks, upper leg, knee, lower leg, and foot/ankle. This pain can be any type of musculoskeletal pain, such as joint pain or soft tissue pain.

Reviewer 2’s comments:

Thanks to the authors for their valuable contributions, I think the manuscript has met the criteria for publication.

Thank you very much for your kind words and approval of our revisions to our earlier work.
